# Beyond Mammography: Sovereignty and Relational Breast Care With Aboriginal and Torres Strait Islander Women

**DOI:** 10.5694/mja2.70245

**Published:** 2026-07-07

**Authors:** Devaleena Das, Jessica Gildersleeve, Amy Thomson, Aunty Gracelyn Smallwood, Lorelle Holland

**Affiliations:** ^1^ Department of Family Medicine and Biobehavioral Health University of Minnesota Duluth Minnesota USA; ^2^ School of Business, Law, Humanities and Pathways University of Southern Queensland Ipswich Queensland Australia; ^3^ School of Education and Creative Arts University of Southern Queensland Ipswich Queensland Australia; ^4^ School of Public Health James Cook University Townsville Queensland Australia; ^5^ ARC Centre of Excellence for Indigenous Futures University of Queensland Brisbane Queensland Australia; ^6^ Faculty of Business, Economics and Law, School of Law University of Queensland Brisbane Queensland Australia; ^7^ Faculty of Health, Medicine, and Behavioural Science, School of Nursing Midwifery and Social Work University of Queensland Brisbane Queensland Australia

**Keywords:** breast neoplasms, culture, mammography

## Abstract

Despite Australia's universal breast screening programs, Aboriginal and Torres Strait Islander women have lower screening participation rates and higher breast cancer mortality. Public health explanations focus on awareness of breast cancer risk and the accessibility of screening, yet these framings often overlook how biomedical screening practices assume a colonial anatomical lens of the body that does not align with Indigenous relational understandings of embodiment. Drawing on Indigenous scholarship, feminist body theory and trauma‐aware care, this article argues that breast screening encounters can become sites where bodily sovereignty and biomedical surveillance intersect. Relational, culturally safe, healing‐centred and sovereignty‐affirming practices, supported by Indigenous‐led initiatives, such as culturally designed screening shawls, can transform screening from a clinical encounter into health care that offers a therapeutic relationship built on trust, dignity and informed consent.

## 1 Introduction: Beyond the ‘Screening Gap’

Breast cancer is the most diagnosed cancer among Aboriginal and Torres Strait Islander women. Yet significant inequities persist across the cancer care continuum. Aboriginal people are more likely to be diagnosed with cancer and less likely to survive at least 5 years after diagnosis compared with non‐Indigenous Australians. Participation in national breast screening programs in Australia remains lower among First Nations women (about 36%) compared with non‐Indigenous women (around 50%) [1]. Aboriginal women are also more likely to be diagnosed at younger ages and at more advanced stages of disease, contributing to poorer survival outcomes and higher rates of mastectomy compared with non‐Indigenous women [2].

Current policy discussions often frame lower participation in breast screening among Aboriginal and Torres Strait Islander women in terms of access, awareness or cultural barriers. At the same time, lower participation reflects a complex interplay of factors, including accessibility of services—particularly for women in regional and remote communities—alongside structural barriers within the health system and the impacts of historically and ongoing culturally unsafe care.

Such framings tend to focus on service uptake and information provision rather than examining the underlying epistemological structure of screening medicine itself. Breast screening programs are built on a particular ontology of the body: the body as a bounded biological object that can be divided into organs, tissues and sites of measurable risk [3]. Within this framework, mammography functions as a surveillance technology designed to identify early pathological change within breast tissue, positioning the breast as a discrete object rather than part of a relational body grounded in Indigenous ways of knowing, being and doing [4]. Population‐based breast screening programs, such as BreastScreen Australia, are implemented through nationally coordinated programs that invite women aged 50–74 years to undergo regular mammography every 2 years for early detection of breast cancer [5, 6]. Importantly, this article does not question the clinical effectiveness of mammography as a screening tool; rather, it examines how breast screening is delivered and experienced, and how these encounters may be strengthened through culturally safe and relational approaches.

Indigenous scholarship has long demonstrated that Aboriginal understandings of health cannot be reduced to the individual body. Health emerges through relations between people, land, ancestors, law and community [7, 8, 9, 10]. In such relational ontologies, the body is not simply a biological organism but a node within a broader network of obligations and meanings. When screening programs encounter bodies that are understood relationally rather than anatomically, a fundamental ontological misalignment emerges. What appears within biomedical discourse as ‘low participation’ may instead reflect a deeper tension between two ways of knowing the body. The aim of this analysis is not to question the clinical value of mammography but to examine how the relational meanings of the body might shape engagement with screening.

## 2 Positionality Statement

This article therefore unites an interdisciplinary collective of Aboriginal and Torres Strait Islander women (A.T., Mandandanji; A.G.S., Birrigubba and South‐Sea Islander woman; L.H., Mandandanji) and researcher and scholarly contributors (D.D., J.G.), who bring their shared expertise in the resistance to colonial architectures that continue to produce unsafe and racialised health care [11, 12, 13]. In doing so, we expose how the breast becomes a site of inequity within these systems, which deny Aboriginal and Torres Strait Islander women access to culturally safe breast screening and sustain the disproportionate and preventable premature mortality associated with breast cancer [14]. Importantly, the lived and professional experiences informing this manuscript are not included as anecdotal supplements to theory; rather, they are understood as embedded, relational forms of knowledge production grounded in community accountability, clinical practice and Indigenous ways of knowing.

In the light of this, improving screening outcomes necessitates more than increased access or enhanced health communication. It requires a shift towards what we call *ontological translation*: clinical practices that engage with Indigenous relational embodiments rather than relying on the assumed universality of biomedical anatomy. By ontological translation, we refer to practices that enable dialogue across different understandings of bodies, health and care without assuming that biomedical understandings are universally experienced or understood. By bringing Indigenous scholarship into dialogue with feminist and decolonial body theory, alongside studies of trauma and healing‐informed practice, we further argue that breast screening must be understood not only as a medical intervention but also as a site where bodily sovereignty and colonial knowledge systems intersect.

The CONSIDER reporting criteria checklist for health research involving Indigenous peoples was completed for this article and can be found in the Supporting Information [15].

## 3 Dreaming, Ceremony and the Relational Breast

This discussion is directly relevant to breast screening, as it highlights how experiences of the body, care and clinical environments are shaped by relational, cultural and ontological frameworks, which in turn influence engagement with and participation in screening programs. BreastScreen encounters are often structured around procedural efficiency and standardisation; however, women arrive at these encounters carrying histories, relationships and embodied meanings that shape how screening environments are experienced. Understanding relational ontologies therefore helps explain not simply whether women attend screening but how screening itself may be interpreted, navigated and experienced.

In many Aboriginal knowledge systems, the body cannot be understood apart from Country, kinship and ancestral law. Aboriginal Law emphasises that identity and humanness emerge through relationships with land, making custodial responsibility the basis of ethical action; by framing land as the source of meaning and the template for social relations, identity and action are grounded in Country, offering a philosophical basis for understanding sovereignty as lived and enacted [16]. The connection between ancestral beings, people and Country is lived through the body, which is formed through inherited ties to place; in this sense, women's bodies themselves express and carry sovereignty [17]. Aboriginal women's bodies have long enacted a sovereign presence shaped by land, ancestors and the stories that bind people to Country, even under conditions of profound colonial violence [18]. Each Aboriginal woman carries the essence of ancestral creative beings, making embodiment itself a continuation of the land's sentience and memory. As a result, everyday actions function as embodied sovereign acts that signal ongoing ownership. Scholars have described this relational ontology through concepts such as Country and Dreaming, which structure the ethical relationships that sustain life.

Dreaming is often mischaracterised within Western discourse as mythology or origin narrative. Indigenous scholars emphasise instead that Dreaming describes an ongoing ontological environment through which land, bodies, ancestors and law remain dynamically connected [7, 8, 9, 10, 19]. These ceremonial practices are not uniform across Aboriginal nations, yet they illustrate broader relational understandings of the body documented across many Indigenous knowledge systems. Within these relational cosmologies, bodily surfaces function as sites through which ancestral knowledge is transmitted. This reflects a conception of humanness in which the body is inherently connected to specific Countries, to human ancestors, to creator beings and to all forms of life [17]. Aboriginal women's embodied responsibilities—care, endurance, resistance and community leadership—are grounded in this same relational ontology and their knowledges emerge through this embodied positioning shaped by gender, Country and ancestral relations [18].

GS's life embodies the relational understandings of the breast described in this section. GS lived and worked as a midwife in her community, witnessing and participating in women's cultural practices that honour the breast as connected to Country, kin and ancestral presence. Her clinical work in Birthing on Country and women's health positioned her at the interface of Indigenous knowledges and Western biomedicine, a bridge she walked with humility and deep cultural grounding. Positioned as a midwife in community, she drew on both her biomedical training and her community's healing knowledges, such as Grandmothers' lore, upholding traditional child‐rearing practices and traditional medicine. Her practice prioritised sovereignty as well as relational and cultural care. This grounding in community, ceremony and relational embodiment offers a powerful real‐world articulation beyond the observational and often abstracted anthropological accounts that follow.

Anthropologist Jennifer Biddle's work on women's ceremonies in Central Australia provides an important lens for understanding the cultural significance of breasts within these relational frameworks [20]. In women's Dreaming ceremonies, breasts are not sexualised body parts nor purely biological organs. Rather, they function as surfaces through which ancestral stories, kinship relations and cosmological responsibilities are enacted. During the ceremony, women paint concentric circles, half‐moons and dot patterns, described as *kuruwarri*, or ancestral designs, onto each other's breasts while singing and dancing Dreaming narratives. The ceremony unfolds collectively: women painting one another, moving together and sharing songs. The breast becomes a site through which ancestral law is remembered and embodied. This ceremonial honouring of the breast stands in stark contrast to the colonial sexualisation and degradation of Aboriginal women's bodies, underscoring the political significance of women's ceremonial embodiment [18]. For Aboriginal people, bodily experiences and memories are not the same, and gender is one of the lenses that helps account for these differences, making women's ceremonial embodiment a distinct mode of sovereign relationality [17].

This ceremonial practice produces what might be described as *intercorporeality*, a mode of embodiment in which the boundaries between individual bodies soften [21]. The breast becomes continuous with land, kinship networks and ancestral presence. Ageing breasts, marked by sagging, trembling and stretchmarks in Western canon, are not stigmatised but honoured as carriers of memory and life force in this ceremonial practice [20]. Within this cosmological framework, breasts are not detachable anatomical units but relational archives. They carry stories, responsibilities and connections that exceed biomedical classification. These perspectives help to explain how breast screening may be experienced differently by Aboriginal and Torres Strait Islander women and why participation is shaped not only by access and information but also by how care aligns with relational, cultural and embodied ways of knowing.

## 4 Colonial Medicine and the Fragmented Body

Western biomedical practice historically developed alongside colonial systems of classification and control. Scientific medicine relied on practices that treated bodies as objects to be examined, mapped and intervened upon [22, 23]. Within this framework, the breast becomes a discrete anatomical structure composed of tissues that can be imaged, measured and assessed for risk. However, feminist body theorists have long argued that bodies cannot be reduced to biological material alone. Bodies are also sites of memory, power and social inscription. Colonial governance frequently targeted Indigenous bodies through surveillance, regulation of sexuality and reproduction, and medical intervention [8, 18]. For many Indigenous communities, the body therefore carries not only biological life but also historical traces of colonial intrusion [8, 18]. Furthermore, since ‘repetition is constitutive’, as Biddle (following Judith Butler) points out [20], the repetition of colonial violence via non‐Aboriginal misunderstandings of the ontology of the body means that each instance itself recalls and reconstitutes those acts of violence.

Trauma scholarship further demonstrates that experiences of violation or forced control over the body can persist as embodied memory, shaping how individuals respond to later encounters involving exposure, touch or surveillance [24]. Trauma is not only psychological but also often somatic, residing in bodily responses to vulnerability, authority and clinical environments. Malik and Hassan's SHARE model emphasises embodied memory as a core construct of understanding the experience of trauma and of trauma recovery within collectivist cultures, and of these as gendered, observing that the body ‘carries the remains of hidden or untold trauma’, and that triggering those memories via the body can also cause physical trauma symptoms [25].

Within this context, mammography screening can become a particularly sensitive site of encounter. The anatomical gaze is foundational to contemporary medical technologies. Mammography, for example, operates by compressing breast tissue between plates to produce radiographic images capable of detecting early pathological changes and is effective as diagnostic technology to prevent premature mortality associated with breast cancer [26]. It is undeniable that mammography remains a powerful and life‐saving diagnostic technology; however, its clinical logic presumes a body that can be abstracted from its cultural and relational worlds. The breast is reduced to tissue density, imaging quality and statistical risk. For patients whose bodily understandings are shaped by relational cosmologies, this abstraction can be profoundly disorienting [27, 28].

This does not mean that screening itself is harmful. Rather, it highlights that clinical technologies are never experienced in isolation from historical and cultural contexts. When the relational meanings of the body are unacknowledged, screening may be perceived not as care but as another moment in which the body becomes an object of institutional scrutiny. Scholarship in culturally sensitive trauma‐informed care, such as the SHARE model [25, 29], therefore reinforces the importance of recognising how power, memory and vulnerability intersect within clinical encounters. Resistance to screening, therefore, cannot be simplistically interpreted as ignorance or non‐compliance. For some women, reluctance or hesitation may emerge from experiences of culturally unsafe care, embodied memories of historical and interpersonal harm or discomfort arising when clinical environments fail to recognise relational understandings of the body. Such responses may reflect epistemic tensions between biomedical and Indigenous understandings of care rather than a rejection of screening itself.

## 5 Research as Ceremony and Relational Care

Indigenous scholar Shawn Wilson proposes that research should be understood as ceremony, a relational practice grounded in accountability, reciprocity and respect [10]. Within this framework, knowledge is not extracted but generated through relationships. Applying this insight into clinical care suggests that screening encounters must begin not with risk statistics but with relational recognition. A relational clinical opening might ask: ‘Before we talk about screening, can you tell me how breast health or bodily care is understood in your Indigenous community or in your own experience?’ Such questions reposition the clinical encounter from information delivery to relational dialogue. This approach also enables what might be described as ontological translation. Instead of presenting mammography solely as a diagnostic technology, clinicians might frame screening in relational terms: ‘This scan helps us look after the strength your breast carries and protect your well‐being for your family and community’. Such translations do not dilute biomedical knowledge; rather, they situate it within culturally meaningful frameworks. Within BreastScreen services, ontological translation could operate at several practical levels: Invitation materials could be co‐designed with communities and incorporate locally meaningful language and imagery; screening appointments could provide opportunities for relational discussion rather than solely procedural preparation; and service environments could include Indigenous staff, culturally familiar spaces and community‐informed practices. Ontological translation therefore refers not only to interpersonal communication but also to how screening systems themselves are organised and experienced. Temporal flexibility is also essential. Health decisions in many Aboriginal communities may involve consultation with family members, Elders or cultural advisors. What may appear to clinicians as delay can instead represent respectful relational decision‐making.

## 6 Cultural Interventions: The Beautiful Shawl Project

Community‐led initiatives demonstrate how culturally grounded interventions can transform clinical encounters. The Beautiful Shawl Project provides screening shawls that have been co‐designed with Aboriginal communities and artists, reflecting place‐based identities and cultural knowledge for women attending breast screening services [28, 30]. This co‐design process is central to the intervention itself, ensuring that the shawls embody connections to family, kinship, community and Country.

Women wear the shawl during mammography and take it home afterwards. This seemingly simple intervention produces several important shifts [31]. First, it restores dignity to an encounter that often requires physical exposure. Second, it introduces cultural presence into a clinical space that might otherwise feel alien. Third, by allowing women to keep the shawl, the act of care extends beyond the clinic into everyday life. The shawl functions not merely as a garment but as a relational object that reconnects screening to community, art and cultural identity. Such initiatives demonstrate that improving participation in screening does not require abandoning biomedical technology: It requires embedding technology within relational, place‐based and culturally meaningful practices. This aligns with broader evidence on the importance of culturally safe health systems for improving outcomes for Aboriginal and Torres Strait Islander peoples. National frameworks, including the Optimal Care Pathway for Aboriginal and Torres Strait Islander people with cancer, emphasise the need for culturally safe, co‐designed and community‐informed approaches across the continuum of care [32]. Initiatives such as the Beautiful Shawl Project are highlighted within this context as examples of how co‐designed, place‐based strategies can build cultural safety, trust and comfort within breast screening services.

These insights have implications beyond a single intervention. At a program level, they suggest that breast screening services, including BreastScreen programs [5, 6], must move beyond a singular focus on access and uptake to consider how screening encounters are structured, experienced and embedded within women's lives. This includes designing services that are relational, culturally safe and co‐developed with communities, and embedding these approaches across the routine delivery of population‐based screening programs. The Optimal Care Pathway for Aboriginal and Torres Strait Islander people with cancer similarly emphasises that culturally safe care is not an adjunct to clinical quality but part of quality itself [32]. Within this framework, initiatives such as the Beautiful Shawl Project demonstrate how co‐designed interventions can move beyond symbolic inclusion to reshape women's experiences of care across the cancer continuum.

## 7 From Compliance to Sovereignty in Clinical Practice

Within BreastScreen services in Australia, these clinical encounters take place within structured, population‐based programs shaped by standardisation, efficiency and participation targets [5]. BreastScreen programs necessarily operate through standardised protocols involving invitations, appointment scheduling, mammography procedures, recall systems and participation metrics. These structures support population‐level effectiveness; however, standardisation alone does not guarantee culturally safe care. Relational approaches therefore should not be understood as alternatives to programmatic screening but as practices that can be integrated within existing systems and workflows.

If screening programs are to support Aboriginal and Torres Strait Islander women's health effectively, they must move beyond a model of behavioural compliance towards bodily sovereignty and relational care, recognising how institutionalised whiteness shapes clinical norms and encounters. This reframing centres Indigenous authority in health and resists the default neutrality of the biomedical gaze that Moreton‐Robinson and Fredericks show to be historically racialised in its design and practice [33, 34, 35]. First, clinical encounters must slow down to allow relational trust to develop. Second, clinicians should recognise that touch is never neutral. Explaining each step of the mammography process and asking consent for positioning can significantly transform the experience of care. Third, screening should be framed as partnership rather than obligation. Instead of emphasising population‐risk statistics, clinicians can invite patients to discuss what health and well‐being mean within their own relational worlds. Fourth, cultural safety must extend beyond individual encounters to institutional structures. Healthcare services must support Indigenous leadership, culturally informed care models and community‐led interventions [14, 36]. Examples within BreastScreen services may include strengthening Aboriginal workforce participation, partnering with local communities during service design, increasing flexibility for family participation where desired, supporting mobile and remote service accessibility and embedding co‐designed cultural practices into routine screening pathways. Finally, clinicians must critically reflect on the assumptions embedded within biomedical language itself. Describing breasts primarily in terms of density, pathology or imaging difficulty can inadvertently reinforce narratives of deficit. Relational language that acknowledges strength, resilience and cultural meaning may foster more respectful care. This has implications for the design and delivery of population‐based breast screening programs, where attention to relational and culturally safe care is as important as access and technology.

## 8 Conclusion

Efforts to increase mammography participation among Aboriginal and Torres Strait Islander women often focus on logistical barriers or public health messaging. Although important, such strategies remain insufficient if they do not address the deeper ontological tensions between biomedical models of the body and Indigenous relational understandings of health. Healing‐centred and relational approaches offer an alternative pathway. By acknowledging the historical and cultural layers that shape bodily experience, clinicians can transform screening encounters from moments of surveillance into opportunities for relational care. This includes adopting a therapeutic orientation that attends not only to technical accuracy but also to emotional safety, cultural presence and the need for clear, supported informed consent, where consent is not a signature but a relational process grounded in dignity and choice. Reframing mammography through relational care does not require abandoning biomedical knowledge. Instead, it requires acknowledging that bodies are never purely biological objects; they are cultural, relational, historicised and storied.

Healthcare systems may not be able to redesign screening programs overnight. However, BreastScreen services and clinicians can begin implementing relational practices within existing structures through culturally safe communication, community partnership, Indigenous leadership and co‐designed care practices. Clinicians can begin immediately by transforming the tone of the encounter: listening relationally, speaking respectfully and recognising that sovereignty begins with how bodies are understood in the clinic. Breast screening participation is not only a behavioural issue but also an ontological encounter between different understandings of the body.

## Author Contributions

**Devaleena Das:** conceptualisation, formal analysis, investigation, methodology, project administration, writing (original draft), writing (review and editing). **Jessica Gildersleeve:** conceptualisation, formal analysis, investigation, methodology, project administration, writing (original draft), writing (review and editing). **Amy Thomson:** conceptualisation, formal analysis, investigation, writing (original draft), writing (review and editing). **Aunty Gracelyn Smallwood:** conceptualisation, formal analysis, investigation, writing (review and editing). **Lorelle Holland:** conceptualisation, formal analysis, investigation, methodology, project administration, writing (original draft), writing (review and editing). We confirm that this work is original and has not been submitted or published elsewhere, and all authors support its submission to the *Medical Journal of Australia* for consideration in the 2026 Special Collection on Indigenous Health.

## Funding

This research was supported by the ARC Centre of Excellence for Indigenous Futures, funded by the Australian Government through the Australian Research Council (Project ID: CE230100027).

## Disclosure

Not commissioned; externally peer reviewed. No AI tool was used in any part of this submission.

## Conflicts of Interest

The authors declare no conflicts of interest.

## Supporting information


**Data S1:** CONSIDER statement.

## Data Availability

This article includes no original data.
