# Supplementary material for: Beyond Mammography: Sovereignty and Relational Breast Care With Aboriginal and Torres Strait Islander Women
Source: Med J Aust. 2026 Jul 7;224(7):e70245. doi: 10.5694/mja2.70245 (PMC13341196; doi:10.5694/mja2.70245)
Supplement: Supplementary file 1 — Data S1: CONSIDER statement. [file MJA2-224-0-s001.pdf]

Supporting Information

Supplementary material

This appendix was part of the submitted manuscript and has been peer reviewed.
It is posted as supplied by the authors.

Appendix to: D. Das, J. Gildersleeve, A. Thomson, et al. Beyond Mammography: Sovereignty and Relational Breast Care with Aboriginal and Torres Strait Islander Women. *Medical Journal of Australia* 2026; doi: 10.5694/mja2.70245.

CONSIDER Statement Template

Guest Editors of the 2025 *Indigenous Health Special Issue* acknowledge the Indigenous expertise that informed the establishment of the CONSolIDated critERia for strengthening the reporting of health research involving Indigenous Peoples (CONSIDER) statement.

Authors should indicate how they have supported ethical publishing and reporting practices by providing the details of the research practices aligned with this publication in accordance with the CONSIDER statement. The reporting should not exceed two pages. This reporting will be published as online supplementary information. Detailed items can be accessed in the publication:

https://bmcmedresmethodol.biomedcentral.com/articles/10.1186/s12874-019-0815-8

**Governance**
This manuscript is Indigenous-led and guided by Aboriginal and Torres Strait Islander scholars, including senior Indigenous leadership. It reflects Indigenous intellectual, cultural, and clinical authority, with knowledge grounded in lived experience, community-informed perspectives, and longstanding engagement in Indigenous health and research.
**Prioritization**
The paper addresses a recognised priority: inequities in breast cancer screening participation and outcomes for Aboriginal and Torres Strait Islander women. It responds to ongoing calls to move beyond deficit-based explanations and to strengthen culturally safe, relational models of care within the Australian health system.
**Relationships (Indigenous stakeholders/participants and Research Team**
The manuscript draws on Indigenous scholarship, cultural knowledge systems, and relational understandings of health. It reflects ongoing relationships between Indigenous researchers, clinicians, and communities, and is informed by Indigenous concepts of relationality, kinship, and connection to Country.

**Methodologies**
As a conceptual Perspective, this work is grounded in Indigenous research paradigms, relational ontology, and decolonising methodologies. It draws on interdisciplinary scholarship including Indigenous knowledge systems, feminist body theory, and trauma-informed care, rather than empirical data collection.
**Participation**
While not an empirical study, the manuscript incorporates Indigenous lived experience, embodied knowledge, and community-informed insights. These perspectives are positioned as legitimate and authoritative forms of knowledge within the analysis.
**Capacity**
The work contributes to strengthening Indigenous-led scholarship and supports the integration of Indigenous perspectives within clinical and public health discourse. It also promotes culturally informed approaches that build capacity within health systems to deliver culturally safe care.
**Analysis and interpretation**
Analysis is grounded in Indigenous epistemologies and critical scholarship examining colonial structures in healthcare. The manuscript reframes screening participation as an ontological and relational issue rather than solely a behavioural or access problem, challenging dominant biomedical assumptions.
**Dissemination**
The manuscript is intended to inform clinicians, researchers, and policymakers, contributing to improved culturally safe breast screening practices. It aligns with broader efforts to translate Indigenous knowledge into health system reform and improved care delivery.
